# Systematic review exploring the relationship between sexual abuse and lower urinary tract symptoms

**DOI:** 10.1007/s00192-022-05277-4

**Published:** 2022-06-25

**Authors:** Caroline Selai, Michael S. Elmalem, Emmanuel Chartier-Kastler, Natalia Sassoon, Sam Hewitt, Maria Francisca Rocha, Larisa Klitsinari, Jalesh N. Panicker

**Affiliations:** 1 UCL Department of Clinical and Movement Neurosciences, Queen Square Institute of Neurology, Queen Square, London, WC1N 3BG UK; 2 Department of Uro-Neurology, The National Hospital for Neurology and Neurosurgery, Queen Square, London, UK; 3 UCL Department of Brain Repair and Rehabilitation, Queen Square Institute of Neurology, Queen Square, London, UK; 4 Department of Urology, Academic Hospital Pitié-Salpêtrière, Medical School, Sorbonne University, AP–HP, 47–83, Bd de l’Hôpital, 75651 Paris cedex 13, France

**Keywords:** Childhood sexual abuse, Childhood trauma, Post-traumatic stress disorder, Lower urinary tract symptoms, Trauma

## Abstract

**Introduction and hypothesis:**

Patients presenting with lower urinary tract symptoms (LUTS) may report a history of sexual abuse (SA), and survivors of SA may report LUTS; however, the nature of the relationship is poorly understood. The aim of this review is to systematically evaluate studies that explore LUT dysfunction in survivors of SA.

**Methods:**

A systematic literature search of six databases, Cochrane Database of Systematic Reviews, MEDLINE, EMBASE, CINAHL, AMED, and PsycINFO, was performed. The last search date was June 2021 (PROSPERO CRD42019122080). Studies reporting the prevalence and symptoms of LUTS in patients who have experienced SA were included. The literature was appraised according to the PRISMA statement. The quality of the studies was assessed.

**Results:**

Out of 272 papers retrieved, 18 publications met the inclusion criteria: studies exploring LUTS in SA survivors (*n*=2), SA in patients attending clinics for their LUTs (*n*=8), and cross-sectional studies (*n*=8). SA prevalence ranged between 1.3% and 49.6%. A history of SA was associated with psychosocial stressors, depression, and anxiety. LUTS included urinary storage symptoms, voiding difficulties, voluntary holding of urine and urinary tract infections. Most studies were of moderate quality. Assessment of SA and LUTS lacked standardisation.

**Conclusions:**

The review highlights the need for a holistic assessment of patients presenting with LUTS. Although most of the studies were rated as being of ‘moderate’ quality, the evidence suggests the need to provide a “safe space” in clinic for patients to share sensitive information about trauma. Any such disclosure should be followed up with further assessment.

## Introduction

Attempted or executed sexual abuse (SA) conducted without consent from the victim can involve penetrative or non-penetrative acts and non-contact [1]. The perpetrator of abuse can range from being a complete stranger to someone familiar to the victim [2] and acts can be committed in private or in public spaces. The prevalence of SA is largely underestimated; however, the results of a recent survey suggests that 1 in 5 women and 1 in 59 men have been exposed to an attempted or completed act of rape during their lifetime [2]. Rates of childhood sexual abuse (CSA) can vary: between 2% and 62% of females and between 3% and 16% of males [3]. The reason for underreporting by victims are manifold, and can include feelings of shame, fear and guilt, a risk of retaliation by the perpetrator [4] and a lack of awareness that forced sexual acts constitute SA [5].

Abuse can have a profound impact on victims, ranging from reduced global functioning levels to lengthened trauma-related symptoms and an increased risk of developing substance abuse [6]. Both male and female victims can report increased rates of depression, anxiety, suicidal ideation and post-traumatic stress disorder (PTSD) [7]. Multiple physical and psychological sequelae have been reported, including anxiety, anger, depression, re-victimisation, self-mutilation, sexual difficulties, substance abuse, suicidality, impairment of self-concept, interpersonal problems, obsessions and compulsions, dissociation and post-traumatic stress responses to somatisation characterised by medically unexplained symptoms [7–11].

Somatisation, functional neurological symptoms and other medically unexplained symptoms can lead to repeated consultations and help-seeking behaviour, which can have significant financial implications in terms of use of health care resources and receipt of financial assistance [12]. Abuse occurring in childhood before the age of 17 (CSA) can result in multiple long-term consequences such as depression, anxiety, poor physical health and risky health behaviours [13]. Furthermore, CSA has been found to be significantly associated with poor outcomes when treating conversion disorders/functional neurological disorder [14].

Urological symptoms are likely to be common amongst survivors of SA. A Dutch study suggested that 2.1% of men and 13% of women seeking urological care may report SA [15]. Many of the physical and psychological sequelae of CSA were found to persist into adulthood [16] and up to one-third of patients attending a gynaecology clinic had experienced CSA [17, 18]. Victims of CSA younger than 6 years old most commonly reported urinary tract infections, daytime incontinence and nocturnal enuresis [19]. SA is likely to be underreported and in the Dutch study, only 15% of participants with a history of SA had disclosed this to their urologist [15]. In a study across five Nordic countries, most women did not disclose a history of SA to their gynaecologist [17]. Seventy percent of Dutch urologists enquired about SA when taking the medical history [20]; however, enquiry rates may vary across specialities and different health care settings.

A recent systematic review and meta-analysis of 38 studies has demonstrated a significant association between a history of sexual assault and developing different gynaecological disorders such as pelvic pain, dyspareunia, dysmenorrhea, abnormal menstrual bleeding and urinary incontinence later in life [21]; however, lower urinary tract dysfunction was not specifically evaluated.

The relationship between SA and LUT dysfunction, however, has been poorly understood. The purpose of this systematic review was to evaluate the reported prevalence of SA, pattern of lower urinary tract symptoms (LUTS) and explore possible associations between SA and LUT dysfunction.

## Materials and methods

The systematic review conformed to the Preferred Reporting Items for Systematic Review and Meta-Analysis (PRISMA) statement and the protocol was registered with the International Prospective Register of Systematic Reviews (PROSPERO; CRD42019122080). A literature search was performed in December 2018 and updated in June 2021 for studies published in the English language without date restrictions in the following databases: Cochrane Database of Systematic Reviews, MEDLINE, EMBASE, CINAHL, AMED, and PsycINFO. The same search strategy (i.e. keywords and inclusion and exclusion criteria) was used for all the databases. The following key words were used: “sexual dysfunction” OR “sexual abuse” OR “adult sexual abuse” OR “sexual trauma” OR “childhood sexual abuse” OR “CSA” OR “sexual maltreatment” OR “rape” OR “sexual offences” OR “sexual harassment” OR “sexual harm” OR “urinary tract” OR “urologist” OR “urological dysfunction” OR “urological symptoms” OR “LUTS” OR “lower urinary tract symptoms” OR “lower urinary tract problems” OR “uroneurology” OR “urethral” OR “genitourinary” OR “urinary frequency” OR “urgency” OR “urinary infection” AND “treatment” OR “management” OR “symptoms”.

Abstracts were imported into bibliography management software (EndNote X8; Thomson Reuters, PA, USA) and were independently evaluated by two reviewers (NS and SH). Studies relevant to the review reporting the prevalence and symptoms of LUTS in male and female patients who have experienced SA were included, whereas experimental studies in animals and studies primarily assessing interstitial cystitis, bladder pain syndrome and pain were excluded. The results of the two reviewers were compared and consensus was achieved by discussion; unresolved differences were reviewed independently (JNP).

Accepted abstracts were retrieved in full text and assessed by the two reviewers (NS and SH), and the following variables were assessed: setting and nature of cohort, definition of SA, assessment of SA, nature of abuse, other types of abuse, nature of LUTS, assessment of LUTS, diagnostic LUTS test and findings, and other co-morbidities. The quality of the studies and risk of bias were assessed using the assessment tool for quantitative studies by the Effective Public Health Practice Project (EPHPP) [22]. Each section was rated by the two reviewers and any discrepancies between scores were discussed and reconciled.

## Results

**Fig. 1:**
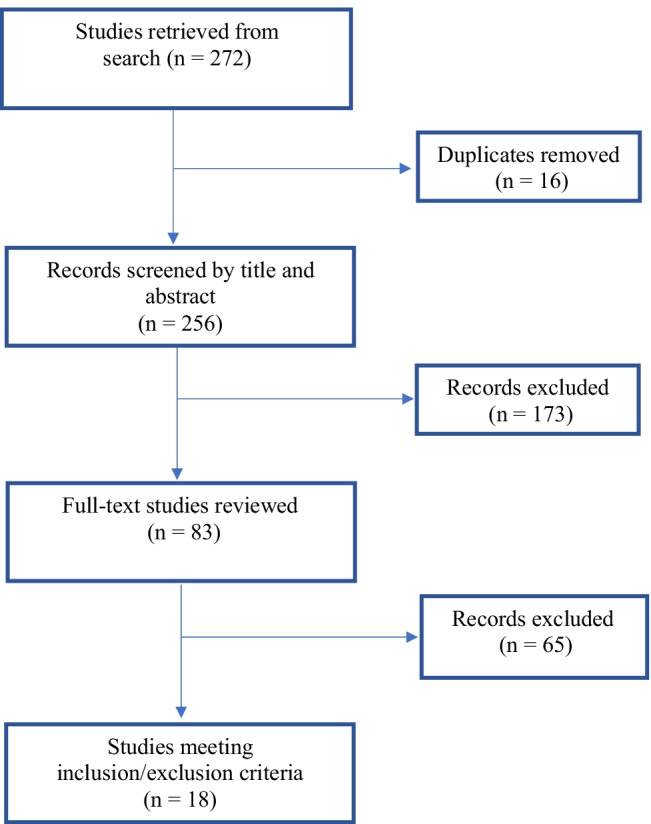
Preferred Reporting Items for Systematic Review and Meta-Analysis flow diagram

The PRISMA flow diagram is presented in Fig. 1. A total of 272 studies were retrieved, and 18 studies met the inclusion criteria: studies exploring LUTS in SA survivors (*n*=2), studies exploring SA in patients attending clinics for their LUTS (*n*=8), and large cross-sectional studies evaluating different health issues including SA and LUTS (*n*=8). The majority of studies were prospective questionnaire-based cross-sectional studies (*n*=13; see Tables 1, 2 and 3). One study was a case–control study [23] and one was longitudinal [24]. The other studies were retrospective, cross-sectional in nature (*n*=3). Fourteen studies were conducted in the US [23–36], 2 in Germany [37, 38], 1 in the Netherlands [39] and 1 in Hong Kong [40].

**Table 1:** Observational studies exploring lower urinary tract symptoms (*LUTS*) amongst sexual abuse survivors (*n*=2)

Study	Setting and cohort	Assessment and completion rate	Definition of SA	Other forms of abuse assessed?	LUT diagnostic tests	Findings	Other comorbidities reported
Davila et al. [25]	Prospective, cross-sectional, questionnaire-based study on childhood SA survivors (*n*=58; mean age 41.5 years) attending support therapy groups (women only). Controls (*n*=51; mean age 48 years) attending a general gynaecology clinic without urological complaints or SA history	CSA and LUTS: non-validated 52-item questionnaire. CSA survivors: female (*n*=58). Controls: female (*n*=51). Completion rate not reported	None	No	None	A total of 72% of CSA survivors vs 22% of controls reported urinary incontinence. Significantly more problems in all (4/4) aspects of stress incontinence (lose with exertion, lose small spurts, lose standing, able to stop flow voluntarily), in (3/8) aspects of urge incontinence (urgency before loss, strong urge with loss, strong urge with loss, leak before reaching toilet) and in (4/6) aspects of voiding dysfunction (difficulty starting stream, slow stream, dribbling/fullness, hold urine until painful) were reported by abuse survivors compared with controls	Diabetes, kidney disease, lumbar disc disease, neurological disease, history of stroke. Significantly more in the abuse survivor group than in controls reported emotional problems, psychiatric conditions, difficulties achieving orgasm and dyspareunia
Postma et al. [39]	Prospective, cross-sectional, questionnaire-based study. Cross-sectional study; 89 young women aged 18–25 years victimised by rape in adolescence; 114 non-victimised age-matched controls. Rape victims: mean age: 20.9 years. Controls: mean age: 20.8 years	Sexual functioning: Dutch version of the FSFI. LUTS: AHPFS-W. Completion rate not reported	Involuntary (attempted or completed) penetration of the victim’s vagina or anus by penis, tongue, fingers, or object, or the victim’s mouth by penis	No	None	Victims 2.7 times more likely to have pelvic floor dysfunction (including LUTS, provoked vulvodynia) than non-victimised controls	PTSD, irritable bowel syndrome, rectal problems, stress. Rape victims had 2.4 times greater likelihood of sexual dysfunction than controls

*CSA* childhood sexual abuse, *FSFI* Female Sexual Function Index, *LUTS* lower urinary tract symptoms, *AHPFS-W* hyperactive pelvic floor scale-women, *PTSD* post-traumatic stress disorder, *SA* sexual abuse

**Table 2:** Observational studies exploring sexual abuse amongst patients attending clinics for lower urinary tract symptoms (*LUTS*; *n* = 8)

Study	Setting and cohort	Assessment and completion rate	Definition of SA	Other forms of abuse assessed?	LUT diagnostic tests	Findings	Other comorbidities reported
Klausner et al. [26]	Prospective, cross-sectional, questionnaire-based study. 121 women attending primary care clinic and referred to specialised LUTS clinic (mean age = 50 years) vs 1,298 controls (mean age 48 years)	Sexual trauma question: “have you ever been forced to have sex against your will?” LUTS: UDI-6, IIQ-7. Completion rates not mentioned	Not fully provided, “sexual trauma”	Emotional abuse, physical abuse	None	Rates of sexual trauma were significantly higher for women in the LUTS clinic (49.6%) (*n*=60/121) than for those in the primary care clinic (20.1%) (*n*=285/1,298). Rates of psychiatric comorbidity were significantly higher for women in the LUTS clinic (64.5%) than for those in the primary care clinic (25.9%). IIQ-7 scores significantly higher in patients with both psychiatric comorbidities and sexual trauma than scores of patients with neither	Psychiatric comorbidities
Lai et al. [29]	Prospective, cross-sectional, questionnaire-based study. Patients (both men and women) in early 50s with OAB (*n*=51) and age-matched healthy controls (*n*=30). Mean age OAB=53.4 years; mean age controls= 54.2years	Childhood traumatic events: Childhood Traumatic Events Scale and Recent Traumatic Events Scale. LUT: ICIQ-UI, ICIQ-OAB, OAB-q, UDI-6, IIQ-7, USS. Completion rates were not mentioned	Not fully provided. Childhood traumatic events occurring prior to age 17	Death of a family member or a close friend, parental divorce or separation, victim of violence, been extremely ill or injured	Clinical assessment of OAB based on AUA guidelines; patients with a positive culture or a post-void residual ≥150 ml were excluded	Childhood sexual trauma more prevalent in OAB patients than in controls, 29.4% (*n*=15) vs 6.7% (*n*=2)	Childhood trauma associated with worse bladder pain, non-urologic pain, poorer mood, higher anxiety, higher somatic symptom burden and higher psychological stress
Jundt et al. [37]	Prospective, cross-sectional, questionnaire-based study. 243 women divided into three groups: OAB (*n* = 85; mean age = 56.3); SUI, (*n* = 101; mean age = 54.6) and control (*n*= 57; mean age = 46.4)	Anonymous, non-validated questionnaire about bladder function and physical/sexual violence. Response rate: 69.4%	Not provided	Physical abuse	N/A	30.6% (*n*=26) of women with OAB had previously been physically or sexually abused, 17.8% (*n*=18) of women with stress incontinence and 17.5% (*n*=10) of the control group had the same history. 85 (34.9%) had OAB, 101 (41.5%) had SUI and the remainder (57, 23.4%) had no LUTS	Not reported
Lutgendorf et al. [27]	Prospective, observational study of 190 women attending a urogynaecology subspecialty clinic. Mean/median age not reported	SA: HITS screen for IPV, and other 30 unvalidated items (demographics, overall health, relationship duration, IPV history and more). Response rate: 75%	Not provided	Physical abuse	N/A	29 women (20%) reported history of forced sex; 4 women (2.8%) positive HITS screen; 39 (27.5%) responded positively to at least one of the HITS items. Lifetime prevalence of physical abuse in 10 women (7%). 23% reported a history of abuse in their family. No statistically significant associations between a positive HITS score and any of the urogynaecology symptoms	Not reported
Bradley et al. [24]	A cross-sectional analysis of a nationwide cohort study. A longitudinal study of 1,702 female veterans (mean age = 31.1years)	SA: questions modified from past national surveys as well as studies on violence in women and female veterans. LUT: computer-assisted telephone interview: the UDI and IIQ-7 (short form). Response rate (48%): 1,702 completed baseline interview. Asked to participate: *n*=3,538	Completed sexual penetration of the vagina, mouth or rectum without a women’s consent, involving the use of force or threat of harm	No	No	Overall, 375 participants (22%) reported overactive bladder, 27% reported prior sexual assault. Female veterans with depression, anxiety or PTSD had two to three times greater odds of OAB symptoms than those without these mental health problems. Increased urinary symptom and functional impact were associated with mental health symptoms and SA	Post-traumatic stress disorder (19%), anxiety (21%), depression (10%)
Ma and Pun [40]	Prospective, cross-sectional questionnaire-based study. Chinese women 18–70 years (mean age=56 years) presenting with urinary symptoms to the Urogynecology Clinic and General Gynecology Clinic, Hong Kong	SA: Modified Abuse Assessment Screen. LUT: ICS definitions and physical examination. Response rate was 96.2%.	Not fully provided “forced sexual activity”	Domestic violence, verbal abuse, physical abuse	No	1.3% reported SA. Abuse survivors (*n*=17): 41.2% OAB symptoms, 53% mixed UI, 5.9% SUI 5.9%. No abuse cohort (*n*=208): 13.9% OAB symptoms, 75% mixed UI, 5.9% SUI 11.1%	Not reported
Cichowski et al. [28]	Retrospective chart review and questionnaire study of 1,899 prospectively recruited female patients (mean age = 54.7) presenting to a urogynaecology clinic	SA: yes/no question enquiring about a history of SA. LUT: interviews using a standardised, physician-administered intake questionnaire. Standardised pelvic examination by the attending physician including the POPQ. Response rate: 66% were asked about a history of SA	Any unwanted sexual activity at any point in time	No	No	SA prevalence 17% (*n*=213). Comparing those with and those without a history of SA, there was no significant difference in prevalence of SUI (57.2% vs 62.4%) and OAB symptoms (50.8% vs 55.9%)	Depression, anxiety, current use of antidepressants/anxiolytics and tobacco use
Komesu et al. [23]	Case–control study, women recruited from general gynaecology and primary care clinics. Mean age of 57 years. 322 participants enrolled	SA: three questions on the BRFSS-ACE Module. LUT: OAB case inclusion: presence of urinary urgency, usually accompanied by frequency and nocturia, with or without urgency incontinence and without other obvious causes. OAB group received the UDI-6 and OABq-SF. Response rate: 322 participants enrolled/427 screened (75%)	Received unwanted sexual touching, forced into unwanted sexual touching; forced to have sex during childhood	The BRFSS-ACE Module incudes abuse (sexual/emotional/physical) and household challenges domains	POPQ examinations ruling out prolapse; urine dipsticks examination ruling out acute urinary tract infections	LUT findings, odds of high ACE occurrence increased two-fold in OAB. 34% (*n*=31) of OAB cases versus 22% (*n*=20) of controls reported sexual ACEs	Compared with controls OAB cases had higher median ACEs (3 vs 1). OAB cases: significantly more fibromyalgia, history of substance abuse, comorbidities (Charlson Comorbidity Index), Depression and Anxiety, OABq symptom score and UDI-6 than controls

*ACE* adverse childhood experience, *BRFSS-ACE* Behavioural Risk Factor Surveillance System Adverse Childhood Experience Module, *CRADI-8* ColoRectal-Anal Distress Inventory, *FSFI* Female Sexual Function Index, *HITS* Hurt–Insult–Threaten–Scream, *ICIQ-OAB* International Consultation on Incontinence Questionnaire Overactive Bladder Module, *ICIQ-UI* International Consultation on Incontinence Questionnaire Urinary Incontinence Short Form, *ICS* International Continence Society, *IIQ-7* Incontinence Impact Questionnaire-7, *IPV* intimate partner violence, *N/A* not available, *OAB* overactive bladder, *OABq* Overactive Bladder symptom and health-related quality of life questionnaire, *OABq-SF* Overactive Bladder Questionnaire-Short Form, *POPQ* pelvic organ prolapse quantification, *PTSD* post-traumatic stress disorder, *SA* sexual abuse, *UDI-6* Urogenital Distress Inventory-6, *USS* Indevus Urgency Severity Scale

**Table 3:** Large cross-sectional studies evaluating different health issues including sexual abuse and lower urinary tract symptoms (*LUTS*; *n* = 8)

Study	Setting and cohort	Assessment and completion rate	Definition of SA	Other forms of abuse assessed?	LUT diagnostic tests	Findings	Other comorbidities reported
Mark et al. [38]	Cross-sectional survey of 730 women (mean age=35.7) presenting to 6 gynaecological and 7 general clinics in Germany	SA: questions on physical and sexual abuse in different periods of life adapted from a previous survey. LUT: non-validated Questionnaire (enquiring about gynaecological symptoms including dysmenorrhoea, vaginal infections, adnexitis, urinary tract infections, menstrual cycle changes and pelvic pain. Response rate 37.6%	Not fully provided	Physical abuse	No	Lifetime prevalence of SA and IPV 52.5% and 28.3% respectively. Urinary tract infections (43.7% seldom, 20.4% frequent/chronic). Urinary tract infections were significantly associated with (frequent/chronic) major physical violence (57.5%), (frequent/chronic) major sexual violence (45.6%) and (frequent/chronic) intimate partner violence (40.5%)	Dysmenorrhoea, (changes in the menstrual cycle, pelvic pain independent of menstrual bleeding, vaginal infections and adnexitis
Link et al. [30]	Analyses were based on data from the Boston Area Community Health survey, a community-based epidemiological study of many different urological symptoms and risk factors comprising a cross-sectional random sample of community-dwelling adults. 5,506 adults, (aged 30–79 years, of which 2,301 men)	SA and LUTS assessed using self-administered (non-validated) questionnaire. Including questions about urinary frequency, urgency and nocturia. Composite measures were created which were “different from the current International Continence Society definitions”. Response rate not reported	Sexual abuse defined as any of the following (unwanted) experiences (and the perpetrator was an adult): exposed sex organs of their body to victim (only included the definition of childhood sexual abuse), threatened to have sex, touched respondent’s sex organs, made respondent touch their sex organs, forced respondent to have sex, or other sexual experiences	Physical and emotional abuse	No	Prevalence of CSA 21.6% (number not reported) and sexual abuse experienced in adolescence/adulthood 19.5 % (number not reported). Urinary frequency, urgency and nocturia positively linked to sexual, physical and emotional abuse. Prevalence of childhood sexual abuse significantly associated with urinary frequency (42.6%, odds ratio 1.74), with urgency (18.1%, odds ratio 1.95) and nocturia (32.8%, odds ratio 1.31). Prevalence of adolescent/adult sexual abuse significantly associated with urinary frequency (40.8%, odds ratio 1.56), with urgency (19.0%, odds ratio 2.09) and nocturia (33.0% , odds ratio 1.31)	Not reported
Nault et al. [31]	Retrospective chart review of consecutive new female patients in their 40s and 50s presenting to a women’s urology centre; (*n*=380; mean age 50 years) divided into four groups according to history: (1) abuse and bullying; (2) bullying but not abuse; (3) abuse but not bullying; (4) neither	SA: unvalidated questionnaires about: sexual health. LUT: Overactive Bladder Questionnaire (OABq-SF), PFDI-20, UDI-6, CRADI-8. Of 380, 338 (89%) answered questions about bullying or abuse	Not provided	No	No	Prevalence of SA: *n*=90 women (24%). Prevalence of bullying: *n*=94 women (24.7%). Women with a history of both SA and bullying had increased overall PFDI-20, POPDI, and UDI-6 scores. CRADI scores increased in patients with a history of SA. OABq-6 and OABq-13 were not significantly different between the groups	History of abuse or bullying or both more likely to suffer from depression, anxiety, IBS, migraines, fibromyalgia and constipation, increased cigarette use. History of abuse and bullying group: increased overall pain and vulvar pain
Bradley et al. [32]	1-year prospective cohort study of female veterans. Eligible women identified through the Defense Manpower Data Center and recruited by mail and telephone. Participants: *n*=1,107. Median age 29 (range 20–67)	SA: lifetime history of sexual assault assessed using questions modified from past national surveys and studies (e.g. National Violence Against Women Prevention Research). LUT: items from UDI-6. Response rate: of 1,702 who completed baseline interview, 1,107 completed the Year 1 interview (65%)	Sexual penetration of the vagina, mouth, or rectum without consent, involving force or threat of harm	No	No	At baseline: OAB was identified in 242 (22%), and 287 (25.9%) reported life-time sexual assault. At 1-year follow-up: 8dence 10.5% and remission rate 36.9%. New onset OAB occurred more often in women with lifetime sexual assault (16% vs 9%, baseline anxiety [21% vs 9%], post-traumatic stress disorder [19% vs 9%])	At baseline: depression (9.2%), anxiety (19.7%), PTSD (17%)
Gibson et al. [33]	Cross-sectional data from National Social Life, Health, and Aging Project, a national area probability sample of older community-dwelling adults (*n*=1,551 older women; mean age = 69)	SA: assessed using the question: “anyone ever made you have sex by using force or threatening to harm to you or someone close to you?” LUT: structured-item questions previously used in epidemiological studies of older women. Response rate: 75.5% overall-weighted response rate	Any lifetime sexual assault	Past-year physical abuse; past-year emotional abuse	N/A	9% (*n*=99) reported sexual assault. Urinary incontinence and other urinary problems reported by 42% and 17% respectively; 42% of sexually active women reported vaginal symptoms with intercourse. Lifetime history of sexual assault not associated with urinary symptoms	42% of sexually active women reported vaginal symptoms with intercourse. 23% reported emotional abuse and 1% reported physical abuse
Boyd et al. 34]	Cross-sectional, multi-ethnic cohort study (*n*=1,999 women; aged 40–80 years; mean age 60.2) enrolled in an integrated health care system	SA:. assessed using the question: “has anyone ever touched sexual parts of your body after you said or showed that you did not want them to, or without your consent?” LUT: structured, interviewer-administered questionnaire items previously validated against a detailed bladder diary. Response rate: 71.7% consented to participate	Not fully provided	Lifetime exposure to physical abuse by an intimate partner, emotional abuse by an intimate partner	N/A	19.7% (*n*=382) reported sexual assault. 45% reported weekly urinary incontinence of any type; 34.5% reported frequent nocturia. 23% stress-type incontinence, 23% urgency-type incontinence, and 35% nocturia. Sexual assault was associated with an increased odds of any bothersome incontinence but not any nocturia outcomes	Emotional IPV associated with increased odds for all urinary symptoms; physical IPV as not associated with any incontinence outcomes, however associated with an increased odds of frequent nocturia. Women with a history of PTSD and depression had increased odds of reporting all urinary symptoms assessed
Lalchandani et al. [35]	Cross-sectional analysis of nationally representative observational data from the National Social Life, Health and Aging Project (*n*= 1,745 women; mean age 71 years)	SA: childhood sexual abuse. One question: “Before you were 12 or 13 years old, did anyone touch you sexually?” LUT: structured questions adapted from other epidemiological studies of older women. Response rate: 79%	Childhood sexual abuse (being touched sexually before the age of 12 or 13)	No	N/A	11.4% (*n*=183) reported childhood SA and 39.2% (*n*=684) reported an unwanted first sexual experience. After adjusting for age, race/ethnicity and education, women with a history of childhood abuse had increased odds of reporting other urinary problems, i.e. voiding difficulties (16.1% vs 27%) but not UI (40.4% vs 45.2%)	Difficulty with ADLs and IADLs and current emotional abuse by family or friends and by their partner. Women with a history of unwanted first sexual experience had increased odds of reporting difficulty with mobility
Geynisman-Tan et al. [36]	Secondary analysis of baseline data obtained from the Symptoms of Lower Urinary Tract Research Network Observational Cohort Study (US). *n*=1,064 (of which 519 men; median age 58.8)	SA: CTES includes sexual and other trauma occurring before age 17. LUT: LUTS tool and PFDI-20 (short form). Completion rate: 95%	Traumatic sexual experience, e.g. rape or molestation	Childhood traumatic events	N/A	25% (*n*=127) of women and 8% of men (*n*=38) reported traumatic sexual experience in childhood. Childhood sexual trauma was significantly associated with greater incontinence severity (adjusted mean difference 4.5 points, 95% confidence interval 1.11–7.88, *p*=0.009). 69% reported at least one childhood traumatic event on the CTES, and 60% of those reported two or more traumas. In women, the number of traumas was associated with worsening PFDI scores, with each additional trauma endorsed increasing the average PFDI score by 4.2	Depression, anxiety and perceived stress, genitourinary pain, bowel symptoms, physical functioning, mobility and sleep disturbance. Approximately half of the effect of Childhood Traumatic Experiences impact score on overall LUTS severity was direct, whereas the other half mediated through the association between trauma and patient’s mental health, i.e. anxiety, depression and perceived stress

*ADLs* activities of daily living, *BRFSS-ACE* Behavioural Risk Factor Surveillance System Adverse Childhood Experience Module, *CRADI-8* ColoRectal-Anal Distress Inventory, *CTES* Childhood Traumatic Events Scale, *FSFI* Female Sexual Function Index, *HITS* Hurt–Insult–Threaten–Scream, *IADLs* independent activities of daily living, *ICIQ-OAB* International Consultation on Incontinence Questionnaire Overactive Bladder Module, *ICIQ-UI* International Consultation on Incontinence Questionnaire Urinary Incontinence Short Form, *IIQ-7* Incontinence Impact Questionnaire-7, *IPV* intimate partner violence, *LUT* lower urinary tract, *OABq* Overactive Bladder symptom and health-related quality of life questionnaire, *OABq-SF* Overactive Bladder Questionnaire-Short Form, *PFDI-20* Pelvic Floor Distress Inventory-20, *PTSD* post-traumatic stress disorder, *SA* sexual abuse, *UDI-6* Urogenital Distress Inventory-6, *USS* Indevus Urgency Severity Scale

### Studies exploring LUTS in survivors of sexual assault

Table 1 summarises the results of two studies [25, 39]. SA was assessed using a non-validated questionnaire including questions about inappropriate unwanted sexual behaviours experienced before the age of 16 [25] or not reported [39]. LUTS were assessed using either a non-validated [25] or validated (Amsterdam Hyperactive Pelvic Floor Scale Women) [39] questionnaire.

### Studies exploring SA in patients attending clinics for their LUTS

Table 2 summarises the results of these studies [23, 24, 26–29, 37, 40]. Four studies used validated scales to assess LUTS: UDI-6 [23, 24, 26, 29], IIQ-7 [24, 26, 29], OABq-SF [23] or a battery of questionnaires (ICIQ-UI, ICIQ-OAB, OABq, USS) [29]. Four studies used non-validated scales or other methods [27, 28, 37, 40].

The prevalence of reported SA ranged from 1.3% [40] to 49.6% [26]. Rates of trauma were significantly higher in patients with LUTS than in control subjects in six studies [23, 24, 26, 29, 37, 40]. SA was assessed using validated scales in three studies: Childhood Traumatic Events Scale and Recent Traumatic Events Scale [29], Modified Abuse Assessment Screen [28], Behavioral Risk Factor Surveillance Scheme BRFSS-ACE Module [23], a non-validated questionnaire [37], a modified previous survey [27], and by a single question [26–28]. The definition of SA differed according to study and included forced sexual activity [27, 40], childhood traumatic events occurring prior to age 17 [29], unwanted sexual activity [28], unwanted sexual touching, forced unwanted sexual touching and forced sex during childhood [23]. A precise definition—complete sexual penetration of the vagina, mouth or rectum without a women’s consent, involving the use of force or threat of harm—was used in only one study ([24]. SA was not defined in two studies [26, 37].

### Large cross-sectional studies evaluating different health issues including SA and LUTS

Table 3 summarises the results of these studies [30–36, 38]. SA was assessed using different methods and only one study used a validated questionnaire, The Childhood Traumatic Events Scale [36]. The prevalence of SA varied greatly between studies, from 9% [33] to 52.5% [38]. A total of 11.4% reported CSA and 39.2% reported an unwanted first sexual experience [35]. The prevalence of CSA was 21.6% and SA in adolescence/adulthood was reported to be 19.5% [30]; 25% (*n*=127) of women and 8% of men (*n*=38) reported traumatic sexual experience [36]. LUTS were assessed using validated questionnaires in only three studies: OABq-SF, PFDI-20; POPDI-6, UDI-6 [31], UDI-6 [32], the LUTS tool and the PFDI-20 [36].

### Assessment of quality of included studies

**Fig. 2:**
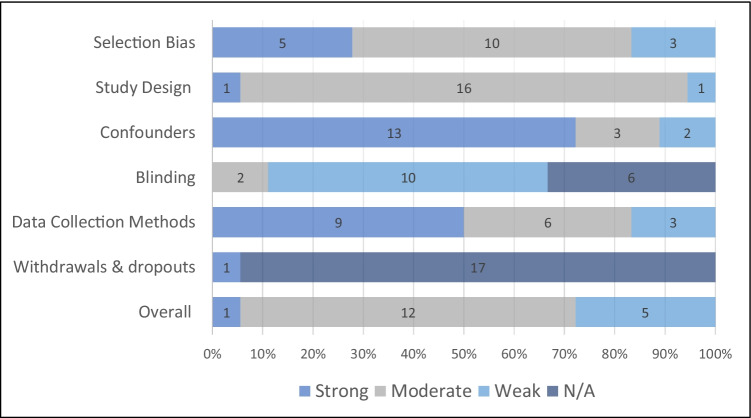
Assessment of quality of included studies using the Effective Public Health Practice Project tool

Using the EPHPP assessment tool, the quality of five studies were rated “weak” [24, 25, 28, 32, 38], 12 studies were rated “moderate” [23, 26, 27, 29–31, 33, 35–37, 39, 40] and only one study was rated “strong” (Fig. 2) [34].

## Discussion

In this review we present a synthesis of 18 studies that explore LUTS in survivors of SA. The wide prevalence of abuse across studies reflects differences in the cohorts studied and heterogeneity in definitions and study designs used. Most studies defined SA broadly as forced or unwanted sexual activity, ranging from the broadest, “unwanted sexual touching” [23] to the narrowest, “complete sexual penetration of the vagina, mouth or rectum without a women’s consent, involving the use of force or threat of harm” [24]. Furthermore, only four studies used a validated scale to assess SA [23, 29, 36, 40], which limited the extent to which the nature, length and severity of abuse could be assessed. The wide prevalence range of SA reported in the studies, from 1.3% [40] to 49.6% [28] may not accurately reflect the true prevalence of SA in patients reporting with LUTS; however, it is somewhat in keeping with the prevalence reported in other cohorts without LUTS [41, 42].

Because of the sensitive nature of SA, there were limits to the extent to which patients could be approached by health care professionals about possible SA. Only 66% of women with pelvic floor disorders were asked about SA [21], whereas in a study exploring physical and SA in patients with an overactive bladder, only women who were not accompanied by a male were approached because of concerns regarding safety [37]. Clinicians would have been reluctant to enquire about SA owing to assumptions that patients may react negatively when questioned [43], lack of familiarity with how to enquire and/or uncertainty about how to proceed if a patient were to disclose SA [20]. In a survey of survivors, more than 70% of abused respondents favourably considered the idea of screening for SA in urological practice [15]. However, patients may not be readily prepared to engage, and over 20% of participants in a study exploring interpersonal trauma and genitourinary dysfunction did not disclose information about sexual assault, more commonly African American and non-partnered women [33]. In a study of Chinese women, which reported the highest response rate of 96%, only 1.3% reported SA and cultural factors of shame and stigma were possible factors responsible for underreporting [40]. Other reasons could include recall bias, disquiet in a public hospital setting, wording of questions about SA and concerns regarding confidentiality.

Lower urinary tract symptoms were variably assessed and urinary storage problems such as urinary incontinence, frequency and nocturia were reported most often. Some patients were reporting incontinence in the context of holding the urine too long until it became painful [25]. Urodynamics testing was not performed in any of the studies. The cause of urinary incontinence was unclear and inclusion of validated questionnaires and possibly urodynamics in future studies would help to understand whether incontinence was due to overactive bladder, stress incontinence or mixed. Establishing dysfunction such as bladder hypersensitivity and/or detrusor overactivity would be critical when tailoring therapeutic strategies for managing these symptoms [44]. Voiding difficulties were less often reported and symptoms reported were pain with urination, hesitancy, slow stream, dribbling, holding urine until painful, incomplete bladder emptying, weak urinary stream and straining to begin urination [25, 33]. Questionnaires such as the UDI-6 do specifically enquire about voiding difficulties; however, only the total score was reported in studies. Urinary retention was not reported and post-void residual volumes were not measured in any of the studies; therefore, the extent of incomplete bladder emptying could not be assessed. Although trauma features in the history of patients presenting with idiopathic urinary retention in men and women [45–47], none of the studies in this review specifically explored urinary retention related to sexual trauma.

Sexual trauma may be one of different types of abuses suffered by individuals, and in these studies emotional and physical abuse [23, 26], violence [29], physical abuse [27, 37], and domestic violence, verbal and physical abuse [40] were reported. Whether other types of abuse contribute to the occurrence of LUT dysfunction is unclear, as an association between emotional abuse and voiding difficulties [35] and urinary incontinence [33] have been reported. Limitations to study designs precluded any meaningful exploration of the association of these different types of abuse with the occurrence of LUTS. The association between trauma and functional somatic syndromes is well established [48, 49] and the stressor response occurring following trauma has been shown to result in physiological changes in body and brain functions that can persist through life and predispose individuals to a range of physical and psychological sequelae.

The age at which SA occurs is also significant; SA occurring during critical developmental periods has been shown to result in profound endocrinological and immunological consequences that may have long-term effects on an individual’s ability to react and respond to illness [50]. Somatic problems such as musculoskeletal pain, ear, nose, and throat symptoms, abdominal pain and gastrointestinal symptoms, fatigue, and dizziness have been found to be more common in adults with a history of childhood trauma than in non-traumatised counterparts [10]. These subjective, medically unexplained physical health problems often persist and present as functional somatic syndromes such as fibromyalgia, chronic fatigue/pain, and irritable bowel syndrome [51]. A recent study found that complex PTSD symptoms mediate the association between childhood maltreatment and trauma and physical health problems. Complex PTSD is associated with a number of psychological sequelae, including hypervigilance, anxiety, agitation, dissociation [52], anger, aggression, self-harm [53], dysregulation in emotion processing, self-organisation (including bodily integrity), relational functioning [54], and psychological interventions that effectively treat symptoms may additionally reduce the risk of physical health problems [55]. Urological symptoms such as OAB are associated with a number of psychiatric conditions such as depression, anxiety and CSA [56].

It is likely, however, that there are different mechanisms responsible for LUTS in survivors of SA. Physical trauma to the perineum and pelvis [57, 58] can result in damage to the regional anatomy. Studies have shown an association between LUTS and anxiety, depression [59–62] and PTSD [63]. Neurobiological mechanisms implicate corticotrophin-releasing factor and serotonergic and dopaminergic systems in the pathogenesis of mood disorders and PTSD, and possible links with LUTS. There is a possibility that adverse life events may lead to neurobiological and physiological changes that increase the risk of both mood disorders and somatic disorders, but that the risk factors may be different [64]. Somatisation may be an adaptive response to psychological distress [65] and although specific symptoms linked to SA have not been consistently identified [66], it is plausible that LUTS may be associated with complex PTSD and a manifestation of somatisation linked to SA; however, this needs to be further explored. Some clinical teams, acknowledging the challenges, are highlighting the need for a multi-disciplinary approach [67]. Notably, duloxetine, a serotonin and norepinephrine reuptake inhibitor (SNRI), that is well established in the treatment of depression and anxiety, has been used with success in the management of both OAB and stress urinary incontinence (SUI) [68, 69].

There were some limitations to this review. Few studies were relevant to the topic, and the overall quality was “moderate”. In the absence of an operational definition for SA, the cohorts differed between studies. Furthermore, a standardised assessment was lacking and therefore the extent of details about types of abuse and their frequency, relationship to the perpetrator, time-frame of abuse, age and impact on childhood development were often missing. A challenge for any research in this area is recall bias, and the wording used in the enquiry about SA and also the setting differed between studies. The extent of rapport and trust between health care professionals and the participants was not assessed; however, these would be critical when exploring such a sensitive topic. Bias in sampling resulting from poor response rates amongst participants approached was not addressed in any of the studies. The assessment of LUTS also differed considerably between studies and therefore the true extent and pattern of LUT dysfunction could not be assessed. Nonetheless, it can be concluded that there exists an association between SA and urinary storage and voiding symptoms.

One major limitation of the review is the low quality and low level of evidence of these 18 studies. Also, the EPHPP does not explore characteristics from each study design that other quality tools can do, such as the Newcastle–Ottawa Scale [70]. There is a need for further research to explain the relation between SA and LUTS. Further, as the studies included in this review were too heterogeneous, a meta-analysis was not performed.

Treatment options, which should take a multi-disciplinary approach, were outside the scope of this review, but, drawing on the current published evidence of treatments for PTSD and complex PTSD, we hypothesise that a proportion of these patients may be helped by trauma-focussed cognitive behavioural therapy and/or other psychotherapeutic interventions.

## Conclusion

The review highlights the need to provide a holistic assessment of patients presenting with LUTS that includes standardised screening for SA in a “safe space” for patients to share sensitive information, and screening for concurrent inter-related factors such as trauma, affective symptoms and somatisation which can impact LUTS. Well-designed studies are required to explore what impact such an assessment may have on the management of LUTS.

## References

[1] Negriff S, Schneiderman JU, Smith C, Schreyer JK, Trickett PK (2014). Characterizing the sexual abuse experiences of young adolescents. Child Abuse Negl.

[2] Breiding MJ (2015). Prevalence and characteristics of sexual violence, stalking, and intimate partner violence victimization—National Intimate Partner and Sexual Violence Survey, United States, 2011. Am J Public Health.

[3] Johnson CF (2004). Child sexual abuse. Lancet.

[4] Sable MR, Danis F, Mauzy DL, Gallagher SK (2006). Barriers to reporting sexual assault for women and men: perspectives of college students. J Am Coll Heal.

[5] Holmes WC (2008). Men’s self-definitions of abusive childhood sexual experiences, and potentially related risky behavioral and psychiatric outcomes. Child Abuse Negl.

[6] Ruch LO, Amedeo SR, Leon JJ, Gartrell JW (1991). Repeated sexual victimization and trauma change during the acute phase of the sexual assault trauma syndrome. Women Health.

[7] Paolucci EO, Genuis ML, Violato C (2001). A meta-analysis of the published research on the effects of child sexual abuse. J Psychol.

[8] Subica AM (2013). Psychiatric and physical sequelae of childhood physical and sexual abuse and forced sexual trauma among individuals with serious mental illness. J Trauma Stress.

[9] Leserman J (2005). Sexual abuse history: prevalence, health effects, mediators, and psychological treatment. Psychosom Med.

[10] Nelson S, Baldwin N, Taylor J (2012). Mental health problems and medically unexplained physical symptoms in adult survivors of childhood sexual abuse: an integrative literature review. J Psychiatr Ment Health Nurs.

[11] Walker JL, Carey PD, Mohr N, Stein DJ, Seedat S (2004). Gender differences in the prevalence of childhood sexual abuse and in the development of pediatric PTSD. Arch Womens Ment Health.

[12] Allanson J, Bass C, Wade DT (2002). Characteristics of patients with persistent severe disability and medically unexplained neurological symptoms: a pilot study. J Neurol Neurosurg Psychiatry.

[13] Neumann DA, Houskamp BM, Pollock VE, Briere J (1996). The long-term sequelae of childhood sexual abuse in women: a meta-analytic review. Child Maltreat.

[14] Van der Feltz-Cornelis CM, Allen SF, Eck V, van der Sluijs JF (2020). Childhood sexual abuse predicts treatment outcome in conversion disorder/functional neurological disorder. An observational longitudinal study. Brain Behav.

[15] Beck JJH, Bekker MD, van Driel MF, Roshani H, Putter H, Pelger RCM (2011). Prevalence of sexual abuse among patients seeking general urological care. J Sex Med.

[16] Warlick CA, Mathews R, Gerson AC (2005). Keeping childhood sexual abuse on the urologic radar screen. Urology.

[17] Wijma B, Schei B, Swahnberg K, Hilden M, Offerdal K, Pikarinen U (2003). Emotional, physical, and sexual abuse in patients visiting gynaecology clinics: a Nordic cross-sectional study. Lancet.

[18] Kaliray P, Drife J (2004). Childhood sexual abuse and subsequent gynaecological conditions. Obstet Gynaecol.

[19] Ellsworth P, Merguerian P, Copening M (1995). Sexual abuse. J Urol.

[20] Beck J, Bekker M, Van Driel M (2010). Female sexual abuse evaluation in the urological practice: results of a Dutch survey. J Sex Med.

[21] Hassam T, Kelso E, Chowdary P, Yisma E, Mol BW, Han A (2020). Sexual assault as a risk factor for gynaecological morbidity: an exploratory systematic review and meta-analysis. Eur J Obstet Gynecol Reprod Biol.

[22] Thomas BH, Ciliska D, Dobbins M, Micucci S (2004). A process for systematically reviewing the literature: providing the research evidence for public health nursing interventions. Worldviews Evid-Based Nurs.

[23] Komesu YM, Petersen TR, Krantz TE, Ninivaggio CS, Jeppson PC, Meriwether KV (2021). Adverse childhood experiences in women with overactive bladder or interstitial cystitis/bladder pain syndrome. Female Pelvic Med Reconstr Surg.

[24] Bradley CS, Nygaard IE, Torner JC, Hillis SL, Johnson S, Sadler AG (2014). Overactive bladder and mental health symptoms in recently deployed female veterans. J Urol.

[25] Davila GW, Bernier F, Franco J, Kopka SL (2003). Bladder dysfunction in sexual abuse survivors. J Urol.

[26] Klausner AP, Ibanez D, King AB, Willis D, Herrick B, Wolfe L (2009). The influence of psychiatric comorbidities and sexual trauma on lower urinary tract symptoms in female veterans. J Urol.

[27] Lutgendorf MA, Snipes MA, O’Boyle AL (2017). Prevalence and predictors of intimate partner violence in a military urogynecology clinic. Mil Med.

[28] Cichowski SB, Dunivan GC, Komesu YM, Rogers RG (2013). Sexual abuse history and pelvic floor disorders in women. South Med J.

[29] Lai HH, Morgan CD, Vetter J, Andriole GL (2016). Impact of childhood and recent traumatic events on the clinical presentation of overactive bladder. Neurourol Urodyn.

[30] Link CL, Lutfey KE, Steers WD, McKinlay JB (2007). Is abuse causally related to urologic symptoms? Results from the Boston Area Community Health (BACH) Survey. Eur Urol.

[31] Nault T, Gupta P, Ehlert M, Dove-Medows E, Seltzer M, Carrico DJ (2016). Does a history of bullying and abuse predict lower urinary tract symptoms, chronic pain, and sexual dysfunction?. Int Urol Nephrol.

[32] Bradley CS, Nygaard IE, Hillis SL, Torner JC, Sadler AG (2017). Longitudinal associations between mental health conditions and overactive bladder in women veterans. Am J Obstet Gynecol.

[33] Gibson CJ, Lisha NE, Walter LC, Huang AJ (2019). Interpersonal trauma and aging-related genitourinary dysfunction in a national sample of older women. Am J Obstet Gynecol.

[34] Boyd BAJ, Gibson CJ, Van Den Eeden SK, McCaw B, Subak LL, Thom D (2020). Interpersonal trauma as a marker of risk for urinary tract dysfunction in midlife and older women. Obstet Gynecol.

[35] Lalchandani P, Lisha N, Gibson C, Huang AJ (2020). Early life sexual trauma and later life genitourinary dysfunction and functional disability in women. J Gen Intern Med.

[36] Geynisman-Tan J, Helmuth M, Smith AR, Lai HH, Amundsen CL, Bradley CS (2021). Prevalence of childhood trauma and its association with lower urinary tract symptoms in women and men in the LURN study. Neurourol Urodyn.

[37] Jundt K, Scheer I, Schiessl B, Pohl K, Haertl K, Peschers UM (2007). Physical and sexual abuse in patients with overactive bladder: is there an association?. Int Urogynecol J Pelvic Floor Dysfunct.

[38] Mark H, Bitzker K, Klapp BF, Rauchfuss M (2008). Gynaecological symptoms associated with physical and sexual violence. J Psychosom Obstet Gynaecol.

[39] Postma R, Bicanic I, van der Vaart H, Laan E (2013). Pelvic floor muscle problems mediate sexual problems in young adult rape victims. J Sex Med.

[40] Ma WSP, Pun TC (2016). Prevalence of domestic violence in Hong Kong Chinese women presenting with urinary symptoms. PLoS One.

[41] Pereda N, Guilera G, Forns M, Gómez-Benito J (2009). The prevalence of child sexual abuse in community and student samples: a meta-analysis. Clin Psychol Rev.

[42] Stoltenborgh M, van Ijzendoorn MH, Euser EM, Bakermans-Kranenburg MJ (2011). A global perspective on child sexual abuse: meta-analysis of prevalence around the world. Child Maltreat.

[43] Peschers UM, Mont JD, Jundt K, Pfürtner M, Dugan E, Kindermann G (2003). Prevalence of sexual abuse among women seeking gynecologic care in Germany. Obstet Gynecol.

[44] Peyronnet B, Mironska E, Chapple C, Cardozo L, Oelke M, Dmochowski R (2019). A comprehensive review of overactive bladder pathophysiology: on the way to tailored treatment. Eur Urol.

[45] Williams GE, Johnson AM (1956). Recurrent urinary retention due to emotional factors; report of a case. Psychosom Med.

[46] Von Heyden B, Steinert R, Bothe HW, Hertle L (2001). Sacral neuromodulation for urinary retention caused by sexual abuse. Psychosom Med.

[47] Selai C, Pakzad M, Simeoni S, Joyce E, Petrochilos P, Panicker J. Psychological co-morbidities in young women presenting with chronic urinary retention. 2019. Available from: https://www.ics.org/2019/abstract/705

[48] Kugler BB, Bloom M, Kaercher LB, Truax TV, Storch EA (2012). Somatic symptoms in traumatized children and adolescents. Child Psychiatry Hum Dev.

[49] Bonvanie IJ, van Gils A, Janssens KAM, Rosmalen JGM (2015). Sexual abuse predicts functional somatic symptoms: an adolescent population study. Child Abuse Negl.

[50] Danese A, McEwen BS (2012). Adverse childhood experiences, allostasis, allostatic load, and age-related disease. Physiol Behav.

[51] Mayou R, Farmer A (2002). Functional somatic symptoms and syndromes. BMJ.

[52] Herman J (1992). Complex PTSD: a syndrome in survivors of prolonged and repeated trauma. J Trauma Stress.

[53] Dyer KFW, Dorahy MJ, Hamilton G, Corry M, Shannon M, MacSherry A (2009). Anger, aggression, and self-harm in PTSD and complex PTSD. J Clin Psychol.

[54] Ford JD (2015). Complex PTSD: research directions for nosology/assessment, treatment, and public health. Eur J Psychotraumatol.

[55] Ho GWK, Karatzias T, Vallières F, Bondjers K, Shevlin M, Cloitre M (2021). Complex PTSD symptoms mediate the association between childhood trauma and physical health problems. J Psychosom Res.

[56] Dolat M, Klausner A (2012). UROPSYCHIATRY: the relationship between overactive bladder and psychiatric disorders. Curr Bladder Dysfunct Rep.

[57] Kim K, Nam J, Choi B (2017). The association of lower urinary tract symptoms with incidental falls and fear of falling in later life: The Community Health Survey. Neurourol Urodyn.

[58] Owusu Sekyere E, Hardcastle T, Sathiram R (2020). Overview of lower urinary tract symptoms post-trauma intensive care unit admission. Afr J Urol.

[59] Coyne KS, Wein AJ, Tubaro A, Sexton CC, Thompson CL, Kopp ZS (2009). The burden of lower urinary tract symptoms: evaluating the effect of LUTS on health-related quality of life, anxiety and depression: EpiLUTS. BJU Int.

[60] Yang YJ, Koh JS, Ko HJ, Cho KJ, Kim JC, Lee S-J (2014). The influence of depression, anxiety and somatization on the clinical symptoms and treatment response in patients with symptoms of lower urinary tract symptoms suggestive of benign prostatic hyperplasia. J Korean Med Sci.

[61] Lung-Cheng Huang C, Ho C-H, Weng S-F, Hsu Y-W, Wang J-J, Wu M-P (2015). The association of healthcare seeking behavior for anxiety and depression among patients with lower urinary tract symptoms: a nationwide population-based study. Psychiatry Res.

[62] Vrijens D, Drossaerts J, van Koeveringe G, Van Kerrebroeck P, van Os J, Leue C (2015). Affective symptoms and the overactive bladder—a systematic review. J Psychosom Res.

[63] Breyer BN, Cohen BE, Bertenthal D, Rosen RC, Neylan TC, Seal KH (2014). Lower urinary tract dysfunction in male Iraq and Afghanistan war veterans: association with mental health disorders: a population-based cohort study. Urology.

[64] Epperson CN, Duffy KA, Johnson RL, Sammel MD, Newman DK (2020). Enduring impact of childhood adversity on lower urinary tract symptoms in adult women. Neurourol Urodyn.

[65] Bae SM, Kang JM, Chang HY, Han W, Lee SH (2018). PTSD correlates with somatization in sexually abused children: Type of abuse moderates the effect of PTSD on somatization. PLoS One.

[66] Iloson C, Moller A, Sundfeldt A, Bernhardsson S (2021). Symptoms within somatisation after sexual abuse among women: a scoping review. Acta Obstet Gynecol Scand.

[67] Cour F, Robain G, Claudon B, Chartier-Kästler E (2013). Childhood sexual abuse: how important is the diagnosis to understand and manage sexual, anorectal and lower urinary tract symptoms. Prog Urol.

[68] Schagen van Leeuwen JH, Lange RR, Jonasson AF, Chen WJ, Viktrup L (2008). Efficacy and safety of duloxetine in elderly women with stress urinary incontinence or stress-predominant mixed urinary incontinence. Maturitas.

[69] Cardozo L, Lange R, Voss S, Beardsworth A, Manning M, Viktrup L (2010). Short- and long-term efficacy and safety of duloxetine in women with predominant stress urinary incontinence. Curr Med Res Opin.

[70] Wells G, Brodsky L, O’Connell D, Shea B, Henry D, Mayank S, Tugwell P. XI Cochrane Colloquium: evidence, health care and culture. Barcelona, Spain; 2003. Evaluation of the Newcastle-Ottawa Scale (NOS): an assessment tool for evaluating the quality of non-randomized studies. http://www.citeulike.org/user/SRCMethodsLibrary/article/12919189.

